# Special Issue “2020 Feature Papers by JDB’ Editorial Board Members”

**DOI:** 10.3390/jdb9020021

**Published:** 2021-06-02

**Authors:** Simon J. Conway

**Affiliations:** HB Wells Center for Pediatric Research, Indiana University School of Medicine, 1044 West Walnut Street, Room R4 W402E, Indianapolis, IN 46033, USA; siconway@iu.edu; Tel.: +1-(317)-278-8780

For this Special Issue “2020 Feature Papers by JDB’ Editorial Board Members,” we present a collection of studies, including original research papers, and review articles by our distinguished editorial board members that focus on advances in understanding multicellular organisms’ growth, differentiation, and remodeling. Significantly, these articles illustrate the diverse range of different animal model systems used by developmental biologists, along with showcasing several state-of-the art methodologies. The articles in this Special Issue underscore the usefulness of different developmental models to help comprehend the mechanisms underlying differentiation of stem and germ cells, organ morphogenesis, growth, and shape, as well as evolutionary developmental mechanisms.

This Special Issue contains five original research articles. The Krumlauf group addressed a fundamental question in Hox gene function during development: namely, the duplication and diversification of vertebrate *Hox* genes. The Hox family of homeodomain-containing transcription factors is a useful model to investigate the evolution of novel DNA binding specificities, as Hox genes have conserved roles in the specification of the anteroposterior (AP) body axis in a wide range of animals, from invertebrates to vertebrates. Significantly, Singh et al. [1] cleverly utilized in vitro differentiation of mouse embryonic stem cells into neuroectodermal cells as their experimental model system to identify Hoxb1 transcriptional targets, using ChIP-Seq. The results are quite surprising in that unlike its closely related paralogous gene Hoxa1, only a small fraction of Hoxb1 targets are co-bound by Pbx and Meis, well known Hox cofactors. In contrast, a substantial portion of its targets are co-bound by Rest, a known transcriptional repressor. These intriguing results provide new mechanistic insights into how vertebrate Hox gene diversify their function after ancestral cluster duplications. The study also highlights the need to gain a deeper understanding of how individual Hox genes regulate their downstream targets, which has the potential to reveal novel mechanisms underlying *Hox* gene diversification. The Conway lab examined the still not fully understood process of mouse spermatogenesis. Using traditional gene expression analysis, Snider et al. [2] determined the temporal expression of previously uncharacterized *Cracd* gene during sperm maturation that coincides with the first wave of meiosis. Additionally, using publicly available single-cell RNA-seq mouse spermatogenesis data, they confirm where Cracd is expressed, and this approach may also indicate which genes it is co-expressed and interacts with. As Cracd expression is detected in round spermatids, the authors hypothesize that Cracd might be a specific marker for the first wave of meiosis and a potential biomarker useful for the diagnosis of azoospermia. This hypothesis was tested in two knockout mouse models presenting with subtle and overt azoospermia phenotypes, enabling the authors to conclude that Cracd might be a useful biomarker of azoospermia phenotype, even prior to an overt phenotype being evident. Similarly, the Beier group provide new data regarding the underlying cause of subtle hippocampal defects previously observed in ENU-generated hypomorphic *Reelin* mutant mice. Ha et al. [3] now show that there are several developmental anomalies in the *Reelin* mutants, specifically that a region of clustered neurogenesis at the edge of the developing infrapyramidal blade is missing. However, they find no large differences in neurogenesis or cell death in the mutant. Instead, they find that the specialized radial glial scaffold, that likely guides these progenitors and newly born neurons into the infrapyramidal blade, is also missing and they suggest that the disruption of this organized neurogenic cluster and the missing scaffold causes the malformation of the infrapyramidal blade. Thus, they suggest that disruption of the neurogenic region is secondary to mispositioning of the cells that initially secrete Reelin, namely the Cajal-Retzius neurons. These three research articles all take advantage of multiple experimental approaches and methods and typify the value of using the mouse in examining differentiation of stem and germ cells and organ morphogenesis within different transgenic models.

A fourth original research article from the Maduro lab describes the evolutional changes in GATA transcription factors in nematodes. GATA factors are an ancient family of transcription factors that mediate processes of development, differentiation, and gene expression in multiple tissues and cell types. Eurmsirilerd and Maduro [4] describe the origin and evolution of GATA factors and mainly focus on regulation via transcription factors (although other factors such as microRNA and epigenetic chromatin regulation also occur). Following bioinformatic identification, alignment, and localization of putative regulatory sequence motifs within GATA factors in numerous nematode species, they present a phylum-wide overview of the GATA factor family in nematodes. These data should encourage future studies to examine tissue and developmental stage-restricted gene expression analysis across the phylum. Additionally, Parra and Johnston [5] use *Drosophila* mutants to address the function of mushroom body defect (Mud), a microtubule-associated protein that contributes to mitotic spindle function during imaginal wing disc growth. Using RNA-seq, the authors show that the lack of Mud alters cell cycle progression and triggers apoptosis with accompanying Jun kinase (JNK) activation. These observations connect spindle-pole formation with transcriptional regulation with JNK/apoptosis signaling with the regulation of formation of extracellular matrix and basal membrane, which is an intriguing result that is relevant for the general question of organ growth and its coordination in development, as well as health and disease.

This Special Issue also contains two review articles. O’Shaughnessy [6] examined the idea that transcriptional bursting (and the formation of ribonucleoprotein (RNP) granules) are a phenomenon that occurs during (and is critical for) epidermal terminal differentiation. Using serval model organisms, including mammals, they summarize recent progress in studies of nascent transcription, cytoplasmic organization of mRNAs, and translation that demonstrate that this process requires mRNA translation during the process of nuclear destruction. As transcriptional noise in the mammalian embryos is highest before cells become committed to a differentiation pathway, this review provides a timely insight into the complexity of cell-fate and lineage commitment transcriptional mechanisms. Chin and Conway [7] outline the function of the mitochondrial Tafazzin gene, which underlies the X-linked human genetic disorder, Barth Syndrome. Significantly, it discusses Tafazzin requirements in yeast, *Drosophila*, zebrafish, mouse, and human induced pluripotent stem cell models. In particular, this review focuses on how these diverse models can provide insight into the developmental basis and prenatal factors that contribute to Barth Syndrome, something that is quite interesting and often overlooked. Both reviews are very comprehensive and feature extensive reference lists. These reviews should stimulate future studies in both of these research areas.

I would like to thank all of our top-notch editorial board members for contributing their work in this inaugural Special Issue. I would also like to thank all our external reviewers for their independent evaluations of the submitted articles, and the editorial staff at the *Journal of Developmental Biology* for their efforts in assembling this exciting Special Issue.
